# HBM4EU Chromates Study: Urinary Metabolomics Study of Workers Exposed to Hexavalent Chromium

**DOI:** 10.3390/metabo12040362

**Published:** 2022-04-18

**Authors:** Lucyna Kozłowska, Tiina Santonen, Radu Corneliu Duca, Lode Godderis, Karolina Jagiello, Beata Janasik, An Van Nieuwenhuyse, Katrien Poels, Tomasz Puzyn, Paul T. J. Scheepers, Monika Sijko, Maria João Silva, Anita Sosnowska, Susana Viegas, Jelle Verdonck, Wojciech Wąsowicz, on behalf of HBM4EU Chromates Study Team, on behalf of Statistical Team

**Affiliations:** 1 Laboratory of Human Metabolism Research, Department of Dietetics, Warsaw University of Life Sciences, 02776 Warsaw, Poland; monika_sijko1@sggw.edu.pl; 2 Finnish Institute of Occupational Health, 00250 Helsinki, Finland; tiina.santonen@ttl.fi; 3 Labotoire National de Santé (LNS), Unit Environmental Hygiene and Human Biological Monitoring, Department of Health Protection, 3555 Dudelange, Luxembourg; radu.duca@lns.etat.lu; 4 Centre for Environment and Health, Department of Public Health and Primary Care, KU Leuven (University of Leuven), 3000 Leuven, Belgium; lode.godderis@kuleuven.be (L.G.); an.vannieuwenhuyse@kuleuven.be (A.V.N.); katrien.poels@kuleuven.be (K.P.); jelle.verdonck@kuleuven.be (J.V.); 5 IDEWE, External Service for Prevention and Protection at Work, 3001 Heverlee, Belgium; 6 QSAR Laboratory Ltd., 80172 Gdansk, Poland; k.jagiello@qsarlab.com (K.J.); t.puzyn@qsarlab.com (T.P.); a.sosnowska@qsarlab.com (A.S.); 7 Laboratory of Environmental Chemoinfomatics, Department of Environmental Chemistry and Radiochemistry, Faculty of Chemistry, University of Gdansk, 80308 Gdansk, Poland; 8 Department of Environmental and Biological Monitoring, Nofer Institute of Occupational Medicine, 91348 Lodz, Poland; beatajan@imp.lodz.pl (B.J.); wojciech@imp.lodz.pl (W.W.); 9 Laboratoire National de Santé (LNS), Department of Health Protection, 3555 Dudelange, Luxembourg; 10 Radboud Institute for Health Sciences, Radboudumc, P.O. Box 9101, 6500 HB Nijmegen, The Netherlands; paul.scheepers@radboudumc.nl; 11 Human Genetics Department, National Institute of Health Dr. Ricardo Jorge (INSA), Toxicogenomics and Human Health (ToxOmics), NOVA Medical School, Universidade Nova de Lisboa, 1169-056 Lisbon, Portugal; m.joao.silva@insa.min-saude.pt; 12 Public Health Research Centre, NOVA National School of Public Health, Universidade NOVA de Lisbon, 1600-560 Lisbon, Portugal; susana.viegas@ensp.unl.pt; 13 Comprehensive Health Research Center (CHRC), 1169-056 Lisbon, Portugal

**Keywords:** biomarkers, biomonitoring, metabolic pathways, early biologic effects, occupational health

## Abstract

Exposure to hexavalent chromium Cr(VI) may occur in several occupational activities, placing workers in many industries at risk for potential related health outcomes. Untargeted metabolomics was applied to investigate changes in metabolic pathways in response to Cr(VI) exposure. We obtained our data from a study population of 220 male workers with exposure to Cr(VI) and 102 male controls from Belgium, Finland, Poland, Portugal and the Netherlands within the HBM4EU Chromates Study. Urinary metabolite profiles were determined using liquid chromatography mass spectrometry, and differences between post-shift exposed workers and controls were analyzed using principal component analysis. Based on the first two principal components, we observed clustering by industrial chromate application, such as welding, chrome plating, and surface treatment, distinct from controls and not explained by smoking status or alcohol use. The changes in the abundancy of excreted metabolites observed in workers reflect fatty acid and monoamine neurotransmitter metabolism, oxidative modifications of amino acid residues, the excessive formation of abnormal amino acid metabolites and changes in steroid and thyrotropin-releasing hormones. The observed responses could also have resulted from work-related factors other than Cr(VI). Further targeted metabolomics studies are needed to better understand the observed modifications and further explore the suitability of urinary metabolites as early indicators of adverse effects associated with exposure to Cr(VI).

## 1. Introduction

Workers with potential exposure to airborne hexavalent chromium (Cr(VI)) carry out work in many industries, including chromium (Cr) metal and Cr metal alloy production and use, electroplating, welding, and the production and use of compounds containing Cr(VI). The construction industry, chromate production, electroplating and grinding of stainless- and acid-proof steel have the greatest number of workers at risk of exposure to Cr(VI) [1]. Absorption by the inhalation route depends on the solubility of the Cr compound, its oxidation state and particle size [2]. Respirable particles can reach the non-ciliated parts of the lungs and, depending on its water-solubility, Cr(VI) may be released and taken up in the circulation until 72 h after exposure (maximum uptake after 6 h) [3]. Dermal exposure to Cr(VI) may occur when the skin is not adequately protected and workers’ skin is directly exposed to liquid forms of Cr(VI), as in electroplating baths [1].

Cr(VI) was classified as a human carcinogen (Group 1) by the International Agency for Research on Cancer [4]. However, the mechanism for carcinogenicity and cytotoxicity of this transition metal is not yet fully understood. In vitro and in vivo studies have shown that when Cr(VI) enters into the body cells, it is reduced to its lower oxidation states Cr(V), Cr(IV) and Cr(III). These reactive Cr intermediates can generate reactive oxygen species (ROS), connected predominantly with the generation of hydroxyl (·OH) radicals and hydrogen peroxide (H_2_O_2_). This ROS generation is related to damage or modification of cellular proteins, lipids and DNA that consequently lead to DNA instability, disturbances in cellular functions and apoptosis (cell death) [5,6,7]. Moreover, the intermediate Cr species formed, i.e., Cr(IV) and Cr(V), are able to form DNA adducts and DNA–protein complexes that may give rise to DNA single- and double-strand breaks that will further contribute to genetic instability and, ultimately, to cancer development [8]. In addition, Cr(III) directly binds to DNA, and especially, ternary Cr-histone-DNA adducts may be important for Cr(VI)-induced carcinogenicity. Cr also disrupts mismatch repair, resulting in genomic instability [9].

Occupational exposure to Cr(VI) compounds has been associated not only with cancers but also with other adverse effects, especially on the respiratory and reproductive systems, skin, kidneys, stomach and liver [10]. Epidemiological evidence indicates that there is a high risk of lung cancer among workers exposed to Cr(VI). There is also evidence suggesting a possible excess of nose and nasal sinus cancer and limited evidence that exposure to Cr(VI) causes stomach or other cancers [11]. Cr(VI) compounds are highly irritating and sensitizing to the respiratory tract, causing, e.g., asthma, bronchitis, rhinitis and ulceration of the nasal mucosa with possible septal perforation [10,12,13]. In the area of research related to fertility potential, Li et al. [14] observed significantly lower sperm counts, higher levels of serum follicle-stimulating hormone and a decreased zinc concentration in the seminal plasma in workers from an electroplating facility compared to the non-exposed group. Dermal exposure to Cr(VI) can be associated with irritant dermatitis related to the direct cytotoxic properties of Cr(VI) and with allergic contact dermatitis as an inflammatory response mediated by the immune system [13,15]. Renal effects after exposure to low doses of Cr(VI) compounds are mainly connected with proximal tubular injury [16] and elevated urinary β2-microglobulin levels, which is an indicator of renal tubular damage [17].

In 2017, the European human biomonitoring (HBM4EU) initiative started as a Joint Programme aimed at standardized and harmonized biomonitoring to study human exposure to chemicals as well as the related health risks, and additionally aimed at the improvement of chemical risk assessment, management and supporting policy making [18], (www.hbm4eu.eu accessed on 10 December 2021). In the scope of HBM4EU, the chromates occupational study was developed. The main aim of the study is to provide EU-relevant data on Cr(VI) internal exposure and early biological effects in occupational settings. A recent article provides details of the overall results and recommendations for the biomonitoring of occupational exposure to Cr(VI) [19]. Some more specific results from this study will be described in follow-up publications that are currently in preparation.

Currently, new markers are being researched, both for exposure (e.g., in blood and urine) as well as early effects (e.g., oxidative stress markers) of Cr(VI). Therefore, efforts have been made to search for new indicators, allowing for the deeper assessment of adverse effects and the risk of health effects [20]. The ultra-high performance liquid chromatography–quadrupole time-of-flight mass spectrometry (UHPLC-QTOF-MS) non-targeted metabolomics method for the quantitative analysis of low-molecular-weight metabolites may be an ideal solution for the identification of significant metabolic differences to further explain the metabolic, physiological, and pathological mechanisms of Cr(VI) toxicity. Thus, such analyses provide a new perspective of understanding the molecular mechanism of metals’ toxicity and prediction of their health effects.

As far as the authors are aware, no such studies have been carried out involving workers exposed to Cr(VI) in several occupational sectors in different European countries. The results of this unique study will allow for the creation of a harmonized platform for further exposure assessment and estimation of health effects from exposure to Cr(VI).

## 2. Results

### 2.1. General Characteristics of the Study Population

The number of workers exposed to Cr(VI) and individuals included in the control groups from each country are presented in Table 1. The study population consisted of 220 male workers exposed to Cr(VI) and 102 male controls.

No significant differences in age, height, body weight and BMI were observed between the entire group of workers exposed to Cr(VI) and the entire control group. On the other hand, the comparison of both groups in terms of smoking status and alcohol consumption showed significant differences (*p* = 0.0001 and *p* = 0.0145, respectively), indicating that a greater percentage of workers exposed to Cr(VI) smoked cigarettes and drank alcohol (Table 2).

**Figure 1:**
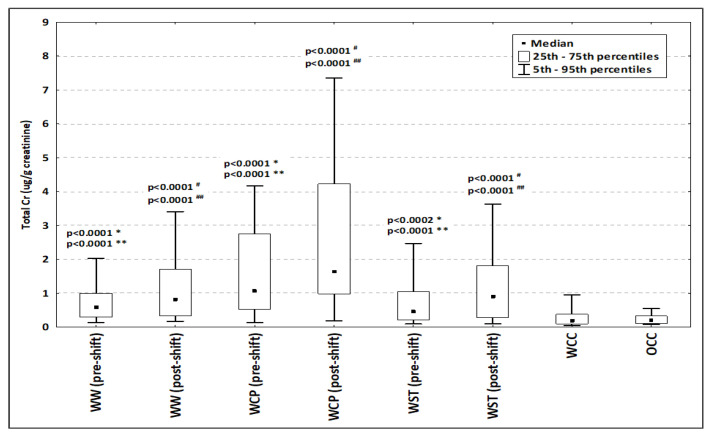
Distribution of urinary total Cr concentration in controls and in exposed workers (pre-shift, post-shift). Abbreviations: WW—welder; WCP—chrome plating; WST—other surface treatment or miscellaneous activities; WCC—within-company controls; OCC—outwith-company controls. * pre-shift vs. WCC; ** pre-shift vs. OCC; ^#^ post-shift vs. both WCC and OCC; ^##^ post-shift vs. pre-shift.

The distribution of urinary total Cr concentration in controls and in workers exposed to Cr(VI) is presented in Figure 1. There were no significant differences in the total Cr concentration in urine between within-company controls (WCC) and outwith-company controls (OCC). Cr concentration in urine in both control groups (WCC and OCC) was significantly lower than in all pre-shift and post-shift groups of workers occupationally exposed to Cr(VI). When comparing the urinary Cr concentrations in pre- and post-shift samples, significantly higher values were observed in post-shift urine samples for all analyzed groups of occupational exposed workers (Figure 1).

### 2.2. Urine Metabolic Changes following Cr(VI) Exposure

**Figure 2:**
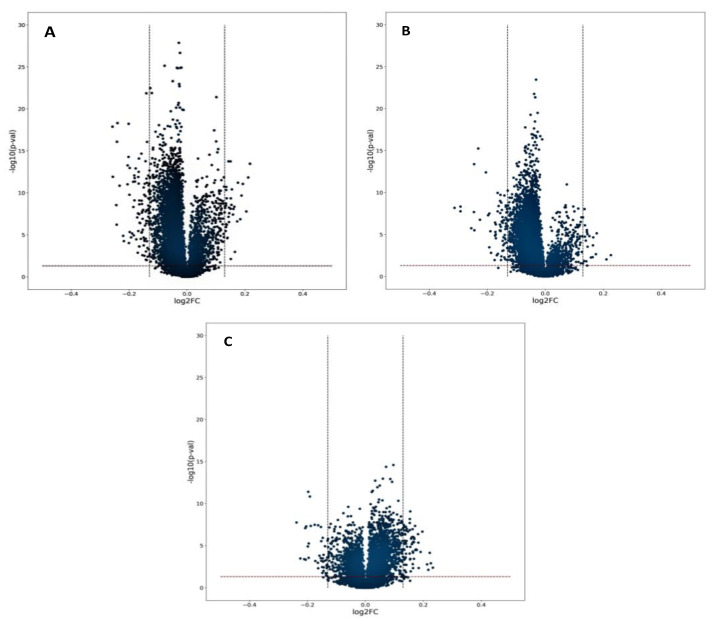
Volcano plots of metabolomic data. The x-axis is a mean log ratio fold-change in the relative intensity of each metabolite between two samples: (**A**)—pre- and post-shift WW; (**B**)—pre- and post-shift WCP; (**C**)—pre- and post-shift WST (gray dotted lines indicate metabolite with |log_2_FC| > 0.13). The y-axis represents the statistical significance of *p*-values of each metabolite. The red line indicates the *p*-value equal to 0.05. Abbreviations: WW—welder; WCP—chrome plating; WST—other surface treatment or miscellaneous activities.

Volcano plots were generated to investigate significant differences between pre- and post-shift samples from each group. Despite Figure 2 indicating that the time of the sample collection (pre- vs. post-shift) significantly affects the metabolites profiles, there were only a few metabolites for which the intensity differed between two samples (metabolite for which |log2FC|>0.13 and *p*-value < 0.05). Most of metabolites were located near the center of the plots, indicating that the majority of metabolites have a relatively low fold-change between compared samples.

**Figure 3:**
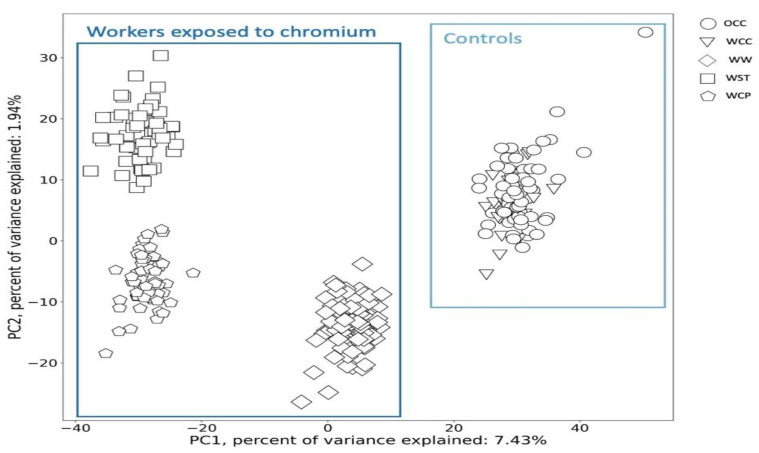
Scatter plot representing the metabolomics profile of the post-shift worker’s urine in the space of PC1 and PC2. Each point marked with a shape corresponds to a different worker’s subgroup. Abbreviations: WW—welder; WCP—chrome plating; WST—other surface treatment or miscellaneous activities; WCC—within-company controls; OCC—outwith-company controls.

In the next step, we explored the distribution of the 322 individuals (220 workers exposed to Cr(VI), considering only their post-shift samples, and 102 controls) in the space of their metabolomic profile (8235 metabolites) using principal component analysis (PCA). The first two PCs together explained 9.37% (7.43% and 1.94%, respectively) of the total variance in the analyzed dataset. PC1 represented 328 metabolites which enabled differentiation between exposed workers and controls. Interestingly, we identified the four major groups of workers along with PC1 (Figure 3): controls (WCC and OCC), welders (WW), other surface treatment or miscellaneous activities (WST) and (chrome plating) WCP. The increase in the PC1 value caused a decrease in the intensity of these crucial metabolites. Looking at the groups on the scatter plot, it could be concluded that workers exposed to Cr(VI) have a different metabolomic profile compared to the control groups, characterized by a higher intensity of the selected 328 metabolites.

PC2 contained two features explaining the differences in the grouping of workers exposed to Cr(VI). The increase in PC values caused a decrease in the intensity of those features, and Figure 3 shows that (according to PC2) they could help to differentiate two groups of workers: WST and WCP. Moreover, the WCP group shows the highest intensity of those two features. After putative annotation, both features turned out to be the same metabolite ubiquinone-2 (ID—HMDB0006709) with *m*/*z* of 336.2174 and retention time of 3.03 min (BEH Amide column with positive ionization mode) and with *m*/*z* of 336.2175 and retention time of 6.59 min (HSST3 column with positive ionization mode).

The PCA analysis revealed 330 features making the largest contribution to the first two principal components (PC1 and PC2) that described the variance in the studied groups of workers and controls.

**Figure 4:**
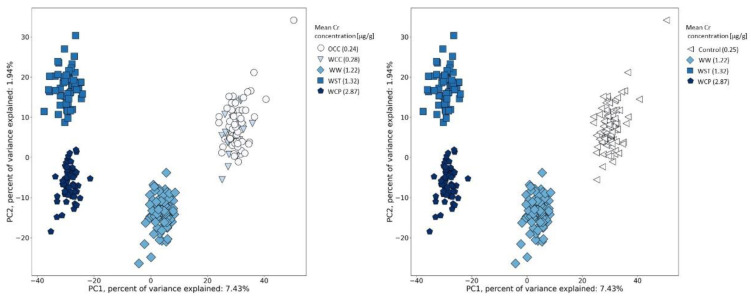
Similarity analysis of metabolomics profiles in urine samples in relation to the arithmetic mean Cr concentration within post-shift workers and controls. Left panel: control groups (WCC—within-company controls; OCC—outwith-company controls) analyzed separately. Right panel: control groups analyzed together (WC—within-company controls and outwith-company controls). Abbreviations: WW—welder; WCP—chrome plating; WST—other surface treatment or miscellaneous activities.

In the next step, we verified whether there was a relationship between the urinary Cr concentration and the metabolomic profile. For this purpose, we colored data points from Figure 3 according to the mean Cr concentration in each of the groups of workers (Figure 4, left panel). Within each of the subgroups of workers, we observed such a relationship. Individuals in the control group were the least exposed to Cr(VI) and had the lowest mean value of metabolite signal intensity. In order to check if the control groups (WCC and OCC) should have been analyzed separately, we performed a Student’s t-test or non-parametric Mann–Whitney test, which showed no differences in these two control groups, and therefore, they could be analyzed together (Figure 4, right panel).

**Figure 5:**
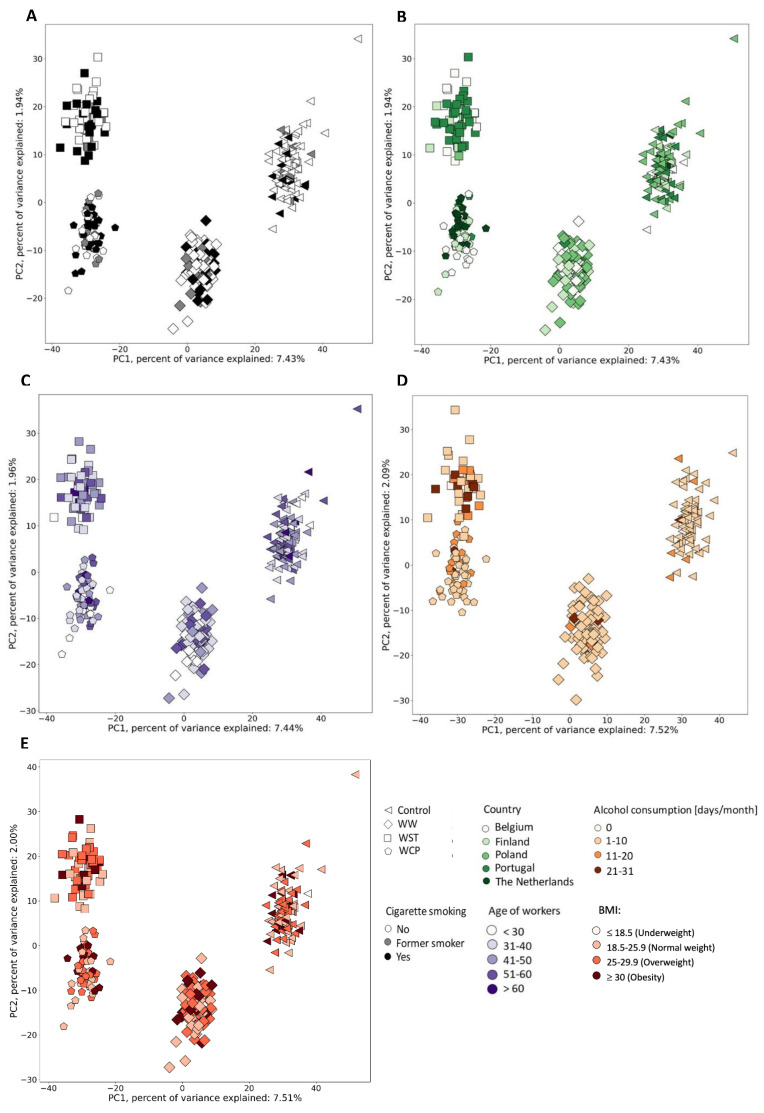
Similarity analysis of metabolomics profiles in urine samples in relation to smoking, alcohol profiles, country, age and body mass index (BMI) within post-shift workers and controls. (**A**)—smoking, (**B**)—country, (**C**)—age, (**D**)—alcohol consumption, (**E**)—BMI. Abbreviations: WW—welder; WCP—chrome plating; WST—other surface treatment or miscellaneous activities; WC—within-company controls and outwith-company controls.

Similarly, we verified whether there was a relation between the metabolomic profile of the workers and their smoking profile, alcohol consumption, their age and body mass index (BMI) as well as the country where the sample was collected. In this case, we colored data points in Figure 5 according to the smoking profile (No, Former smoker, Yes) (Figure 5A), according to the country of sample origin (Figure 5B), age (Figure 5C), alcohol consumption (here, we considered the days per month with declared alcohol consumption) (Figure 5D) and BMI (Figure 5E). In these five examples, no trends were observed between and within the subgroups of workers.

Compared to the WCC and OCC groups, workers exposed to Cr(VI) had a different metabolomic profile, which was characterized by a higher intensity of 328 features. From these features, we putatively annotated 110, and some of them were the same metabolite detected with different ionization modes or on two types of chromatographic columns. For one metabolite, ubiquinone-2 (HMDB0006709, oxidative phosphorylation pathway), we observed that its intensity was significantly higher in the control group compared to the group of workers exposed to Cr(VI). The list of putatively annotated metabolites is presented in Supplementary Materials Table S1. These results are a first exploration of the effects of Cr(VI) exposure on changes in metabolic pathways with some interesting leads for follow-up studies.

**Figure 6:**
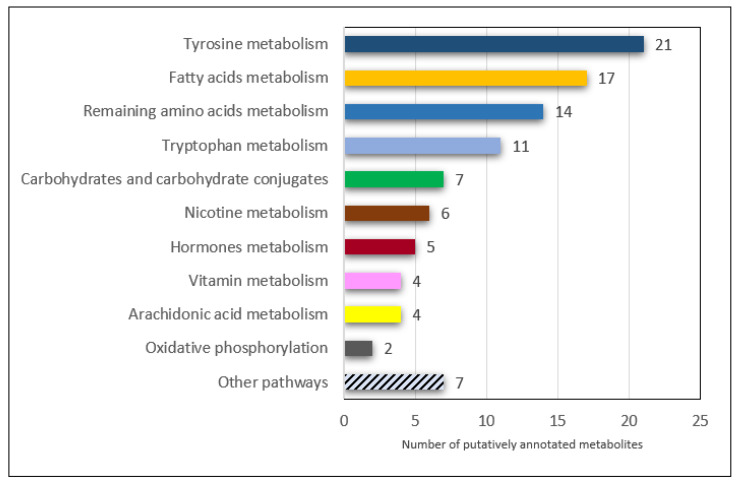
Metabolic pathways with numbers of putatively annotated metabolites in urine samples, the signal intensity of which was significantly higher in the post-shift groups of workers with occupational exposure to Cr(VI) compared to the control group (within-company controls and outwith-company controls).

The ascribed metabolites are related to the following metabolic pathways: tyrosine, tryptophan and other amino acids; fatty acids and arachidonic acid; carbohydrates and carbohydrate conjugates; vitamin, nicotine and hormones; and oxidative phosphorylation (Figure 6).

At least two metabolites were annotated in each of these identified pathways. In the remaining seven pathways, only one metabolite was annotated in each, and the metabolites were as follows: methane, purine, ribonucleoside, phenylpropanoid, short-chain hydroxy acids and derivatives, creatine, limonene and pinene. The greatest number of metabolites was putatively annotated in the pathways of amino acid metabolism, such as the metabolic pathways of tyrosine, tryptophan and other amino acids (alanine, arginine, aspartate, lysine, phenylalanine, alpha amino acids and dipeptides). In the pathways of tyrosine and tryptophan metabolism, in addition to the metabolites belonging to the degradation subpathways of these amino acids, we annotated metabolites that were associated with the metabolic subpathways of neurotransmitters, such as dopamine (10 metabolites: DL-dopa, 3-methoxytyrosine, dopamine, dopamine glucuronide, N-acetyldopamine, 3,4-dihydroxyphenylacetaldehyde, 3-methoxytyramine, 3,4-dihydroxybenzylamine, 1,2-dehydrosalsolinol and 3,4-dimethoxyphenylethylamine), norepinephrine and epinephrine (four metabolites: epinephrine, epinephrine glucuronide, vanylglycol and 3,4-dihydroxyphenylglycol) and serotonin (three metabolites: 5-hydroxy-L-tryptophan, 5-hydroxytryptophol and formyl-5-hydroxykynurenamine). In the pathway of fatty acid metabolism, acyl glycines were putatively annotated, which are minor metabolites of fatty acids (12 metabolites: 2-furoylglycine, 3-methylcrotonylglycine, capryloylglycine, isobutyrylglycine, suberylglycine, phenylpropionylglycine, 2-methylhippuric acid, nicotinuric acid, 6-hydroxyphenylpropionylglycine, 3-hydroxyoct-1-enoylglycine, 6-hydroxyoct-1-enoylglycine and 3-hepteneoylglycine) and another 5 metabolites belonging to chemical sub classes: fatty acids and conjugates, medium-chain fatty acids, fatty acid esters and phenol esters.

In the subclass carbohydrates and carbohydrate conjugates, we putatively annotated seven compounds from the following pathways: the starch and sucrose, galactose, glycosides, pentose and glucuronate interconversions and the pentose phosphate pathway.

**Figure 7:**
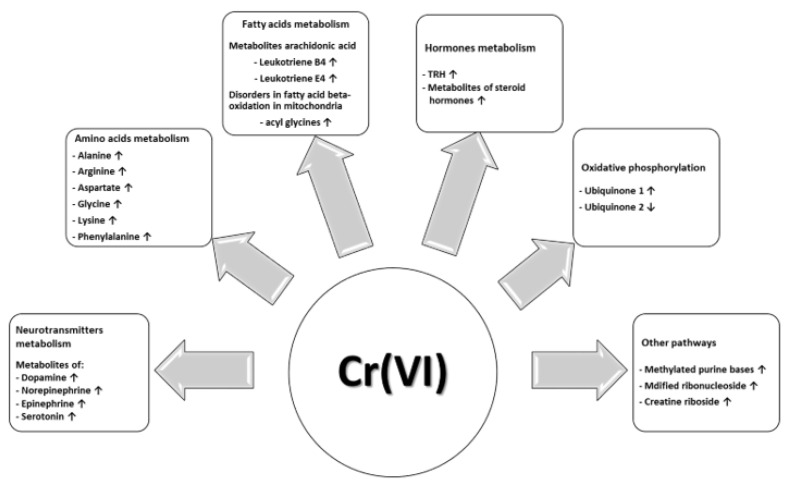
Potential relationship between Cr(VI) exposure and biological metabolic outcomes based on the results obtained from post-shift urine samples in the groups of workers. Abbreviations: ↑—significant increase; ↓—significant decrease.

The remaining pathways in which at least two metabolites were annotated were: nicotine metabolism (six metabolites: 2’-Hydroxynicotine; 5’-hydroxycotinine; anatabine; cotinine N-oxide; norcotinine; nornicotine), vitamin metabolism (four metabolites: pyridoxamine; pyridoxine; N1-(alpha-D-ribosyl)-5,6-dimethyl-benzimidazole; 6-methylnicotinamide), hormones metabolism (five metabolites: thyrotropin-releasing hormone; 2-methoxyestrone; 6-ketoestriol; androsterone; epitestosterone sulfate), arachidonic acid metabolism (four metabolites: 12-keto-tetrahydro-leukotriene B4; 12-oxo-10,11-dihydro-20-COOH-LTB4; 20-carboxy-leukotriene B4; 20-oxo-leukotriene E4) and oxidative phosphorylation (two metabolites: ubiquinone-1; ubiquinone-2). The proposed relationship between Cr(VI) exposure and their metabolic outcomes are summarized in Figure 7.

### 2.3. Candidate Biomarkers of Occupational Exposure to Cr(VI)

**Figure 8:**
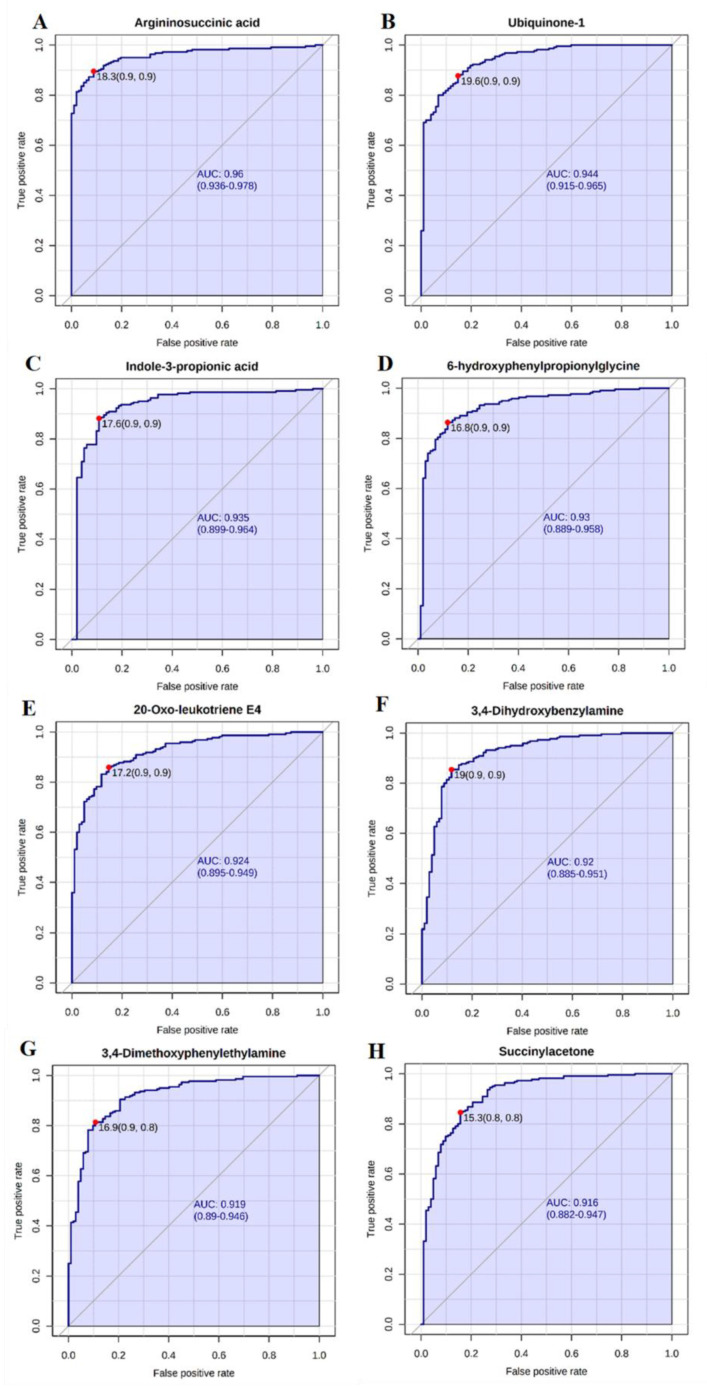
(**A**–**H**). ROC curve generated from the spectral data to identify urine metabolomic biomarkers indicating probable Cr(VI) exposure. Notes: (**A**) argininosuccinic acid, (**B**) ubiquinone-1, (**C**) indole-3-propionic acid (**D**) 6-hydroxyphenylpropionylglycine, (**E**) 20-oxo-leukotriene E4, (**F**) 3,4-dihydroxybenzylamine, (**G**) 3,4-dimethoxyphenylethylamine, (**H**) succinylacetone. Abbreviations: AUC—area under the curve; ROC—receiver operating characteristic.

The clear separation of the group of workers exposed to Cr(VI) in comparison to the controls observed in the space of the two first PCs (Figure 3) suggests different urinary profiles of metabolites are observed in subgroups of workers depending on the industrial sector. As a consequence, there are metabolites that can be considered as potential biomarkers of Cr(VI) exposure. Therefore, we performed the classical univariate receiver operating characteristic (ROC) curve analysis (Figure 8A–H) to identify the key metabolite changes suitable for distinguishing workers from controls. For this purpose, we only applied those metabolites that were significant for the first PC considering that this PC was responsible for separating the workers exposed to Cr(VI) and the controls.

We observed that: argininosuccinic acid (area under the curve—AUC = 0.959; *p* = 1.04 × 10^48^), ubiquinone-1 (AUC = 0.943; *p* = 1.24 × 10^49^), indole-3-propionicacid (AUC = 0.934; *p* = 1.05 × 10^27^), 6-hydroxyphenylpropionylglycine (AUC = 0.927; *p* = 7.03 × 10^44^), 20-oxo-leukotriene E4 (AUC = 0.923; *p* = 8.99 × 10^43^), 3,4-dihydroxybenzylamine (AUC = 0.919; *p* = 7.41 × 10^40^), 3,4-dimethoxyphenylethylamine (AUC = 0.918; *p* = 1.47 × 10^42^) and succinylacetone (AUC = 0.914; *p* = 1.01 × 10^35^) are potential biomarkers for Cr(VI) exposure with an AUC higher than 0.9 (intensities of all these metabolites are significantly higher in the case of workers exposed to Cr(VI) in comparison to controls).

## 3. Discussion

We provided the first explorative metabolic profiling of workers occupationally exposed to Cr(VI) using collected urine samples analyzed using liquid chromatography coupled to high resolution mass spectrometry. In order to detect as many metabolites as possible, we used the newest sample extraction techniques and two types of chromatographic columns: for polar, semi-polar and non-polar metabolite separations. This allowed us to detect many metabolites, which significantly differed in abundancy depending on the studied worker’s subgroups with exposure to Cr(VI), (WW, WCP and WST) and controls (OCC and WCC). Urine samples for metabolomics studies were obtained from five countries that participated in the HBM4EU chromates study (exposed workers *n* = 220, controls *n* = 102). The greatest number of putatively annotated metabolites belonged to pathways of amino acids, especially tryptophan and tyrosine metabolism, fatty acids and sub pathway arachidonic acid metabolism, as well as the metabolism of carbohydrates and carbohydrate conjugates, vitamins, hormones, nicotine and oxidative phosphorylation. Below, we further discuss the observed changes in each of the pathways in more detail.

### 3.1. Tyrosine, Tryptophan and Other Amino Acid Metabolism

In workers occupationally exposed to Cr(VI), we observed a significantly higher signal intensity from metabolites of neurotransmitters such as dopamine, norepinephrine, epinephrine and serotonin. Dopamine is a precursor of epinephrine and norepinephrine, acts as a major transmitter in the brain, is involved in regulating a variety of physiological activities, plays an important immunomodulatory function and is found in the cardiovascular system and intestine [21,22]. In high levels, dopamine acts as a metabotoxin (an endogenous metabolite that causes adverse health effects), causing neurotoxicity (the disruption of neural tissue and/or its function) [23]. Serotonin function is connected with regulations of many physiological functions, e.g., food intake, the sleep–wake cycle, memory, cognition and emotion, but dysfunctions in the serotonergic system are linked to the development or progression of mental disorders [24]. We observed a higher-intensity signal for 3,4-dihydroxyphenylacetaldehyde—a metabolite of oxidative deamination of dopamine, which generates a free radical and stimulates the activation of mitochondrial permeability transition and consequently leads to neuron death [25]. Wu et al. [26] analyzed multiple metal exposures (titanium, vanadium, chromium, manganese, iron, cobalt, nickel, copper, zinc, arsenic, cadmium and lead) in Chinese electroplating workers, and correlations were observed in the urinary concentrations of twelve main neuroactive metabolites related to dopamine, serotonin and kynurenine pathways. In exposed workers (detailed descriptions of the studied group and performed activities, e.g., how close workers were to the chrome bath, are lacking), the urinary concentration of vanadium, manganese, cobalt, nickel, copper, zinc, arsenic and cadmium were significantly higher than in the control group, but unexpectedly, Cr concentrations in the Chinese electroplaters were at a similar level as in the control group. In the Chinese workers, higher urinary levels of tryptophan, 5-hydroxyindole acetic acid, kynurenine, 3, 4-dihydroxyphenylacetic acid; 3-methoxytyramine, homovanillic acid, norepinephrine and epinephrine were observed. Additionally, the urinary concentration of Cr correlated positively with concentrations of epinephrine and norepinephrine. Changes in the monoamine neurotransmitter metabolism were also observed in a population environmentally exposed to arsenic [27]. Our study indicated that in workers with a higher Cr urinary concentration, there is a similar profile regarding monoamine neurotransmitter metabolism as in people exposed to arsenic, but in workers exposed to Cr(VI), we detected far more metabolites from these pathways.

In the secretion of hormones and neurotransmitters, a diurnal fluctuation is observed, referred to as the circadian clock [28]. Cortisol, serotonin, adrenaline and dopamine rhythms peak in the morning [29,30]. In our study, there was some variation in the sample collection time: in exposed workers, all post-shift samples were collected in the afternoon; in controls, about half were morning samples. We consider that the time of sample collection did not have an impact when taking into account that, e.g., serotonin and dopamine levels should actually be higher in the morning than in the afternoon—thus, at most, this difference in sampling times would rather result in higher than lower levels in controls, at least in the case of these neurotransmitter levels.

Workers occupationally exposed to Cr(VI) also had higher-intensity signals from many amino acids’ metabolisms such as alanine, arginine, aspartate, glycine, lysine, phenylalanine and other amino acids, compared to controls. Among others, high-intensity signals from N-acetyl-L-tyrosine, succinylacetone and cystathionine ketamine were observed. It has been shown that transition metals such as Cr(VI) can be involved in the oxidative modification of amino acid residues in proteins as well as some oxidative modification in free amino acids. Histidine, proline, arginine and lysine residues have been identified as major targets for oxidation [31,32]. For example, histidine residues are converted to aspartate or asparagine residues; proline residues are converted to glutamic acid, pyroglutamic acid and γ-glutamicsemialdehyde residues; arginine residues are converted to γ-glutamicsemialdehyde residues [32]. N-acetylated amino acids such as N-Acetyl-L-tyrosine can be released by an N-acylpeptide hydrolase during proteolytic degradation [33]. Many N-acetylamino acids, including N-acetyltyrosine, are classified as uremic toxins [34] at high concentrations. Succinylacetone, an abnormal tyrosine metabolite, can be created by the oxidation of glycine and is suspected to be genotoxic. If present in sufficiently high levels, succinylacetone can act as an acidogen, an oncometabolite and a metabotoxin [35]. A high content of succinylacetone in urine was observed, for example, in a rat model of irritable bowel syndrome in comparison to the control group [36]. In human peripheral blood polymorphonuclear leukocytes, it was found that pre-incubation with cystathionine ketimine has a priming effect on superoxide generation [37]. 

### 3.2. Arachidonic Acid and Other Fatty Acids Metabolism

In the occupationally exposed workers, we observed a higher-intensity signal from metabolites of arachidonic acid—leukotriene B4 (LTB4) and leukotriene E4 (LTE4). Arachidonic acid is an omega-6 polyunsaturated fatty acid that is found abundantly in the human inner surface of the cell membrane. Esterified arachidonic acid is hydrolyzed by phospholipase A2 to its free form and further metabolized by cyclooxygenases, lipoxygenases and cytochrome P450 enzymes to eicosanoids (bioactive mediators), including, inter alia, to leukotrienes: LTB4, LTC4, LTD4 and LTE4 [38,39]. When eicosanoids are overexpressed, they can induce inflammation and take part in the development of inflammatory, cardiovascular and autoimmune diseases as well as cancers [39,40,41]. Cysteinyl leukotrienes (LTE4) activating their receptors on target cells increase the permeability of small blood vessels in the respiratory tract and enhance the secretion of mucus [42]. It was also shown that Cr(VI) can lead, for example, to health problems such as the incomplete perforation of the nasal septum, pneumonitis, bronchitis and asthma and has been associated with bronchial cancer [3,13,43]. In a study on HepG2 cells exposed to Cr(VI), Zhong et al. [44] observed severe cellular stress acting as an indicator of inflammatory activity. Our observations of higher-intensity signals from metabolites of LTB4 and LTE4 may indicate the enhancement of the inflammatory response to Cr(VI) exposure. The increased concentration of LTB4 was also observed during nickel exposure [45].

Out of the 17 fatty acid metabolites with higher intensity in the workers’ groups, 12 were acyl glycines, which are minor metabolites of fatty acids. The impairment of the mitochondrial β-oxidation of fatty acids is connected with the accumulation of non-oxidized acyl-CoA esters, the activation of alternative pathways of oxidation, such as ω-oxidation and the synthesis of acyl-derivatives such as acyl-carnitines, acyl-glycines and acyltaurines. The concentration of these metabolites in body fluids is measured during diagnostics of disorders associated with fatty acid beta-oxidation in mitochondria [46]. It has also been shown that mitochondrial dysfunction may be linked to Cr(VI) exposure and is associated with the inhibition of mitochondrial respiratory chain complex I and III as well as the induction of electron transfer chain dysfunction, which consequently leads to the perturbation of mitochondrial respiration and redox homeostasis [47,48,49].

### 3.3. Hormones Metabolism

In groups of workers exposed to Cr(VI), upregulated thyrotropin-releasing hormone (TRH) and four metabolites of steroid hormones were also putatively annotated. The main role of TRH is the regulation of TSH expression, which in turn induces the synthesis and release of thyroid hormones [50]. TRH biosynthesis is regulated not only by triiodothyronine (T3), [51,52], but also, for example, by steroids [51,53]. TRH also interacts with other neurotransmitters such as 5-HT (5-hydroxytryptamine), dopamine and noradrenaline [54,55]. Research in the field of neurotransmitter interactions with TRH showed that noradrenergic, serotonergic, cholinergic and GABAergic systems may be influenced by TRH or mediate some of its actions [56]. Therefore, the upregulation of neurotransmitter and steroid hormone metabolites observed in this study can be also connected to higher-intensity signals from TRH. In a rat model, the effect on the hypothalamus, pituitary and thyroid gland structure and function was analyzed following two acute intraperitoneal doses of potassium dichromate administration. In rat glands, such changes were observed as: excessive DNA fragmentation and the death of cells, a decrease in serum free thyroxine (fT4) and free triiodothyronine (fT3) levels and an increase in serum thyroid-stimulating hormone (TSH) concentration [57]. In the study developed by Li et al. [58] on Bufo gargarizans (B. gargarizans) embryos treated with a high dose of Cr(VI), disruptions to the thyroid endocrine pathway and lipid metabolism were linked to reduced thyroid hormone activity (via the decreased type II iodothyronine deiodinase and increased type III iodothyronine deiodinase expression), which impaired fatty acid synthesis and were also linked with the decreased mRNA expression of thyroid hormone receptors β, which led to the acceleration of fatty acid β-oxidation. In the above-mentioned animal model studies, high doses of Cr(VI) were used, so it is difficult to translate these results into a population of workers exposed to low doses of Cr(VI).

Nascimento et al. [59] investigated the possible association between the exposure to xenobiotics (pesticides and metals) and thyroid dysfunction in children living in a rural community in southern Brazil. They observed that blood levels of Cr, Mn, Hg and Pb, as well as hair Pb levels, were positively correlated with TSH concentrations and negatively associated with fT4 levels in the low-exposure period. In high-exposure periods, in comparison to low-exposure periods, higher serum concentrations of thyroid-stimulating hormone and lower concentrations of fT4 and fT3 were observed. Disruption in the thyroid hormones pathway during exposure to heavy metals was also observed in the Korean National Environmental Health Survey in the general population. In this study, urinary Hg concentration was negatively associated with total T3 in both males and females. Additionally, a positive association between Cd exposure and thyroid autoantibodies was demonstrated in males [60].

Regarding metabolites of steroid hormones, the present study observed upregulations related to androsterone, epitestosterone sulphate and metabolites of estrogens (2-methoxyestrone, 6-ketoestriol). Androsterone is a metabolite of testosterone and dihydrotestosterone (DHT) and is a weak androgen with a potency that is approximately 1/7 that of testosterone [61,62]. Epitestosterone action is connected with competitive binding to androgen receptors, antigonadotropic activity as well as with the inhibition of testosterone biosynthesis and its reduction to dihydrotestosterone [63]. Several studies performed on animal models indicated that chronic exposure to Cr(VI) leads to potential reproductive toxicity connected with oxidative stress in the testis, semen and epididymis [64,65,66] and with infertility development [64,65,66,67]. Cr is accumulated in testicular tissue [68] and remains there for a long time [69]. In rats with prenatal exposure to Cr(VI), the vacuolation of mitochondria in Leydig cells [70] was also observed. A recent study suggested that prenatal exposure to Cr(VI) also decreased the expression of StAR protein in Leydig cells, which consequently led to the limited availability of cholesterol that is converted in mitochondria to pregnenolone [71]. Exposure to Cr(VI) also downregulated the steroidogenic enzymes and their specific activities involved in cholesterol conversion to progesterone, pregnenolone, progesterone, androstenedione and testosterone, respectively. Additionally, adverse effects on the expression of steroidogenic receptors for gonadotropins, prolactin, androgens and estrogens were also observed. These changes led to a decrease in testosterone biosynthesis, an increased serum concentration of luteinizing hormone, follicle-stimulating hormone, 17β-estradiol as well as a decrease in testosterone and prolactin in F1 rats with prenatal exposure to Cr(VI) [71].

### 3.4. Oxidative Phosphorylation

In the pathway of oxidative phosphorylation, two metabolites were annotated, of which the intensity signal derived from ubiquinone-1 was significantly higher in the group of workers exposed to Cr(VI) in comparison to controls, but the intensity signal from ubiquinone-2 was significantly lower. Ubiquinone-2 was the only metabolite whose signal intensity was significantly lower in the group of workers exposed to Cr(VI). Ubiquinone-2 is an analogue of ubiquinone-10 [72]. Coenzyme Q10 (CoQ10) is a redox compound with two bioactive states: oxidized (ubiquinone) and reduced (ubiquinol), and both forms are bioactive [73]. The fully oxidized form, ubiquinone, is commonly referred to as ubiquinone 10, Q10 or CoQ10, and the fully reduced form, ubiquinol, is referred to as ubiquinone-1, QH2 or CoQH2. Ubiquinone-1 is an intermediate in the synthesis of ubiquinone 10. CoQH2 inhibits protein, DNA and lipid oxidations, and this last effect has been most thoroughly studied. CoQH2 was observed to inhibit the peroxidation not only of cell membrane lipids but also reduce lipids present in the circulation [74]. The action of CoQ10 is much better known and is connected with a number of cellular functions, especially within mitochondria [75]. During oxidative phosphorylation, CoQ10 plays a role as an electron carrier in the mitochondrial electron transport chain. Additionally, as cofactor of dihydroorate dehydrogenase, CoQ10 is involved in the regulation of the mitochondrial permeability transition and metabolism of pyrimidines, fatty acids and mitochondrial uncoupling proteins [75]. CoQ10 is also involved in the antioxidant protection of cellular membranes [76] and in the regeneration of vitamins C and E [77]. In addition, CoQ10 has a role as a mediator of inflammation and a role in processes such as metabolism of cholesterol, sulphide and amino acid [78]. CoQ10 has been shown to directly affect the expression of a number of genes [79].

There are only a few studies on the effects of heavy metals exposure on CoQ10. Abdallah et al. [80] have shown that total CoQ10 levels in serum, brain, liver and kidney tissues in lead-treated rats were significantly lower than in the control group and that CoQ10 is susceptible to environmental toxins, which may cause CoQ10 deficiency. In L-02 hepatocytes after Cr(VI) exposure, changes such as: the downregulation of the gene expression of electron transfer flavoprotein dehydrogenase as well as a reduction in the coenzyme CoQ10 level, mitochondrial biogenesis (presented by mitochondrial mass) and mitochondrial DNA copy number were observed. Moreover, many unfavorable changes such as mitochondrial damage and apoptosis were observed, which were characterized by reactive oxygen species accumulation, caspase-3 and caspase-9 activation, decreased superoxide dismutase and ATP production, increased methane dicarboxylic aldehyde content, mitochondrial membrane depolarization and mitochondrial permeability transition pore opening, increased Ca2+ levels, cytochrome c release, decreased anti-apoptotic B-cell lymphoma-2 family proteins expression, and significantly elevated pro-apoptotic Bax proteins expression [44]. Given the broad spectrum of CoQ10 activity, the changes in signal intensity that we observed, especially from ubiquinone-2, may be associated with many metabolic pathways not only related to oxidative phosphorylation.

### 3.5. Nicotine, Vitamins and Other Pathways

In the group of workers exposed to Cr(VI) versus the control group, a higher-intensity signal from six metabolites derived from nicotine was observed. This observation is consistent with the results of questionnaire studies, which showed a higher percentage of smokers in the group of workers exposed to Cr(VI). Smoking did not have a statistically significant effect on the signal intensity of other metabolites. Higher signal intensities were also observed for metabolites of vitamin B_2_, B_6_ and niacinamide (a form of vitamin B_3_), which may indicate their higher consumption, and this can have a beneficial effect on the reduction/prevention of Cr(VI) toxicity. Several studies have shown the beneficial effects of certain vitamins and minerals in reducing Cr(VI) toxicity [81]. In rats, vitamin B_6_ pretreatment and simultaneous treatment with Cr(VI) administration reduced lipid peroxidation in the liver tissue [82].

Among the metabolites belonging to the other pathways, attention should be paid to 7-methylguanosine, 1,7-dimethylguanosine and creatine riboside. 7-methylguanosine is an endogenous, methylated purine base that is more abundantly present in malignant cells. The formation of certain DNA adducts leads to DNA damage such that proper and complete replication cannot occur, resulting in genetic instability [83,84,85]. 1,7-dimethylguanosine is a modified ribonucleoside formed in tRNA enzymatic methylation at high concentrations of methylating agents and is linked to carcinogenesis. A high serum concentration of modified ribonucleosides was observed in patients with chronic renal failure [86,87]. Creatine riboside, a conjugate of creatine and ribose, is considered as a diagnostic marker for lung cancer [88]. Taking into account the results of studies showing the relationship between the increased concentrations of these three metabolites, it seems that the higher signal intensity from these compounds may indicate the initiation of unfavorable changes in the group of workers exposed to Cr(VI). 

### 3.6. Candidate Biomarkers of Occupational Exposure to Cr(VI)

We annotated eight potential biomarkers of occupational exposure to Cr(VI) with an AUC higher than 0.9. These biomarkers belong to the following pathways: arachidonic acid metabolism (20-oxo-leukotriene E4), oxidative phosphorylation (ubiquinone-1), fatty acids metabolism (6-hydroxyphenylpropionylglycine) as well as tyrosine (succinylacetone, 3,4-dihydroxybenzylamine, 3,4-dimethoxyphenylethylamine), tryptophan (indole-3-propionic acid) and other amino acids’ metabolism (argininosuccinic acid). The discussion of the first three metabolites is presented in the previous sub-sections. The other two are related to the tyrosine pathway 3,4-dihydroxybenzylamine and 3,4-dimethoxyphenylethylamine and are metabolites of the monoamine neurotransmitter metabolism. 3,4-dihydroxybenzylamine is known as an alternative substrate for dopamine and is a precursor to epinephrine and norepinephrine [89]. 3,4-dimethoxyphenylethylamine is an analogue of dopamine with a substitution of the hydroxy groups with methoxy groups. This metabolite exhibits little known bioactivity but has been shown to have neurotoxic effects, especially in the nigrostriatal system and among dopaminergic neurons; it seems to be an inhibitor of the mitochondrial complex I [90,91] and is a risk factor for Parkinson’s disease associated with metal exposure [92,93].

Indole-3-propionic acid is a product of tryptophan formed by bacteria in the gastrointestinal tract [94] and is a marker for the presence of Clostridium sporogenes in the gut [95]. Indole-3-propionic acid has been shown to have an antioxidant effect [96], and it is suggested that its higher urinary concentration may reflect an additional compensatory mechanism counteracting the negative effects of acute inflammation. On the other hand, recent preliminary studies suggest that a misbalanced human tryptophan metabolism with higher urinary indole-3-propionic acid concentration reflects multiple sclerosis disease activity and severity [97].

Argininosuccinic acid is a precursor to fumarate in the citric acid cycle via argininosuccinate lyase. Argininosuccinic acid is particularly toxic to astrocytes and neurons in organotypic cell cultures by decreasing their viability [98]. In the knockout mice model of argininosuccinic aciduria, increased brain levels of nitrite, nitrate, and nitrotyrosine indicated that oxidative/nitrative stress are involved in neurotoxicity [99,100]. Argininosuccinic acid also markedly disturbs redox homeostasis in the brains of developing rats, and it is feasible that high brain levels of this metabolite may potentially activate pathways inducing reactive species generation [101].

These eight biomarkers with the highest AUC indicate that the main disturbances observed in workers exposed to Cr(VI) were associated with: LTE4 overexpression, alterations in oxidative phosphorylation connected with higher-intensity signals from ubiquinone-1, the impairment of the mitochondrial β-oxidation of fatty acids connected with the accumulation of non-oxidized acyl-CoA esters and the activation of alternative pathways of oxidation such as the synthesis of acyl-glycines, disturbances in monoamine neurotransmitter metabolism, the oxidative modification of the amino acid residues and with the excessive formation of amino acid metabolites such as indole-3-propionic and argininosuccinic acid.

Kuo and co-workers [102] reported on non-targeted metabolomics carried out in a group of 35 male welders employed in a shipyard and 16 male controls. Welders were exposed to different concentrations of Cr, nickel, and manganese particles, and urinary metabolomics analysis showed higher levels of glycine, taurine, betaine/TMAO, serine, S-sulfocysteine, hippurate, gluconate, creatinine, and acetone and lower levels of creatine. In this study, a different analytical technique (1H NMR spectroscopy) and sample preparation was used, and in addition, workers welded different materials; these factors may have resulted in a different metabolite profile compared to our study.

### 3.7. Strengths and Limitations of the Study

Undoubtedly, the advantage of the conducted research is the large number of workers exposed to Cr(VI) who performed welding, chrome plating and surface treatment activities. Certainly, it would be possible to distinguish a few more subgroups, but this would entail a significant reduction in the number of workers in each subgroup. The gathering of such a large number of workers exposed to Cr was possible as part of the international cooperation within the HBM4EU chromates study. In this project, particular attention was paid to the standardization of methods such as: sample collection, storage and transport, and protocols for these procedures have recently been published [19].

The limitation of this study is the fact that it only included men. It was not possible to collect urine samples from women performing the above-mentioned activities related to Cr(VI) exposure, as these are typically male professions. Additionally, factors other than Cr(VI) exposure could have contributed to the observed changes, e.g., occupational exposures to other metals and exposures to particles (e.g., in welding) or pre-existing medical conditions such as hypertension or diabetes (no information was collected on this). An additional weakness of our study is the limited standardization of urine sample collection times, and therefore, we are not sure how circadian rhythm has influenced our findings.

## 4. Materials and Methods

### 4.1. Study Population

The HBM4EU chromates study involved a large group of 399 workers exposed to Cr(VI) [19]; the metabolomics study included a subset of these Cr(VI)-exposed workers from five of the participating countries: Belgium, Finland, Poland, Portugal and the Netherlands. Questionnaire data and urine samples were obtained from a cohort of 220 male workers undertaking activities resulting in occupational exposure to Cr(VI) such as: WW, WCP and WST. The WST group was heterogeneous and included: painting or removing Cr(VI) paints and stainless steel production (Belgium), thermal spraying (Finland), Cr(VI) painting and related jobs (Portugal) and surface treatment by sandblasting (Poland). The information gathered from questionnaires on age, height, body weight, smoking and alcohol consumption were self-reported and not objectively tested. Company and worker recruitment, ethical approvals and specific questionnaire questions are detailed elsewhere [103]. Briefly, the worker inclusion criteria for the metabolomics study were as follows: occupational exposure to Cr(VI), sex—male with ages ranging from 18 to 70 years and present at work during the planned period of the study. In addition, there was a workers’ population of 102 male controls not involved in these kinds of activities which formed a within-company control group, for example, office staff (WCC, *n* = 33) and a group of *n* = 69 outwith-company controls from other companies with no activities associated with Cr(VI) exposure (OCC).

### 4.2. Urine Collection and Cr Analysis

A single spot urine sample was collected from the control group workers at any time during their working week. Two spot urine samples were collected from the workers occupationally exposed to Cr(VI)—the first before the start of the shift at the beginning of the work week (pre-shift), and the second at the end of the shift towards the end of the work week (post-shift). Urine sample collection, handling, storage and transfer covered within the HBM4EU Chromates study were carried out according to “Standard operating procedure for urine sampling, including sample storage and transfer” [103]. All urine samples were collected between October 2018 and December 2020. Urinary creatinine concentrations were measured, and Cr results were normalized to creatinine [104]. Laboratories analyzing Cr concentration in urine samples successfully passed Interlaboratory Comparison Investigations rounds within the HBM4EU Quality Assurance program [105]. Cr concentration in urine was measured using inductively coupled plasma–mass spectrometry (in Poland, Belgium, Finland and the Netherlands) and using graphite furnace atomic absorption spectrometry (in Portugal).

### 4.3. Sample Preparations for Untargeted Metabolomics

Urine samples from exposed workers and from a control group were randomized and divided into 7 batches. Before analysis, samples were extracted using two assays [106]. Assay 1 was used to extract semi-polar and non-polar metabolites, and the procedure steps were as follows: 100 μL of urine (thawed on ice) and 300 μL of ice-cold solvent (50:50 water:methanol with internal standards: Benzoyl-D5 and L-phenylalanine 3,3-D2) were mixed, vortexed (120 s) and centrifuged (21,000 *g*, 20 min, 4 °C). An amount of 200 μL of the supernatant was aliquoted into a low-recovery-volume HPLC vial. Assay 2 was used for the extraction of polar metabolites and was performed as follows: 100 μL of urine (thawed on ice) and 300 μL of ice-cold solvent (50:50 acetonitrile: methanol with internal standards: Benzoyl-D5 and L-phenylalanine 3,3-D2) were mixed, vortexed (120 s) and centrifuged (21,000 *g*, 20 min, 4 °C). An amount of 200 μL of the supernatant was aliquoted into a low-recovery-volume HPLC vial. Pooled “quality control” (QC) samples were prepared by mixing equal aliquots of urine of 10 μL from all samples. Each analytical batch included: 10 samples for system equilibration, 76–79 urine samples from controls/workers, 9 QC samples, 2 blanks and 2 test mixes. During the analytical run, the QC sample was injected into every ten experimental samples to monitor system consistency.

### 4.4. Metabolomics Analysis

The chemicals applied were: formic acid LC-MS grade Chem-LAB NV (Zedelgem, Belgium), acetonitrile and methanol LC-MS grade Avantor Performance Materials Poland S.A. (Gliwice, Poland), ammonium formate 99.99% Sigma-Aldrich (St. Louis, MO, USA), Benzoyl-D5, 98% and L-phenylalanine 3,3-D2 98% Cambridge Isotope Laboratories, Inc. (Andover, MA, USA). Ultra-high-purity water was prepared with a R5 UV Hydrolab system (Wislina, Poland). Sodium formate calibration solution and leucine encephalin lock mass solution (Waters, UK) were prepared according to the manufacturer’s specifications.

Analysis of the urine samples was performed using a Waters Acquity™ Ultra Performance LC system (Waters Corp., Milford, MA, USA) connected to a Synapt G2Si Q-TOF mass spectrometer (Waters MS Technologies, Manchester, UK) equipped with an electrospray (ESI) source (Waters, Manchester, UK). For semi-polar and non-polar metabolite separations (Assay 1), an ACQUITY UPLC HSS T3 column (1.8 μm, 2.1 × 100 mm) with an ACQUITY UPLC HSS T3 1.8 um, VanGuard pre-column 2.1 × 5 mm (Waters, Milford, MA, USA) were applied. The injection volume was 2 μL (both in ESI+ and ESI-), and separation was performed at 0.3 mL/min and 40 °C. The gradient mobile phase was a mixture of 0.1% formic acid in water (A) and in acetonitrile (B). The optimized gradient elution procedure (both in ESI+ and ESI-) was as follows: 0–0.5 min 1% phase B; 0.5–2 min from 1% to 10% B; 2–4 min from 10% to 20% B; 4–5 min from 20% to 30% B; 5–6 min from 30% to 50% B; 6–8 min from 50% to 99% B; 8–11 min 99% B; 11.0–11.5 min from 99% to 1% B; 11.5–15.0 min 1% B.

For polar metabolite separations (Assay 2), an ACQUITY UPLC BEH Amide column (1.7 μm, 2.1 × 100 mm) with an ACQUITY UPLC BEH Amide 1.7 um, VanGuard pre-column 2.1 × 5 mm (Waters, Milford, MA, USA) were used. The injection volume was 2 μL (both in ESI+ and ESI−), and separation was performed at 0.5 mL/min and 35 °C. Mobile phase A: 95% acetonitrile/water (10 mM ammonium formate, 0.1% formic acid); mobile phase B: 50% acetonitrile/water (10 mM ammonium formate, 0.1% formic acid). The optimized gradient elution procedures (both in ESI+ and ESI−) were as follows: t = 0.0 min, 1% B; t = 1.0 min, 1% B; t = 3.0 min, 15% B; t = 6.0 min, 50% B; t = 7.5 min, 95% B; t = 8.5 min, 99% B; t = 10.0 min, 99% B; t = 10.5 min, 1% B; t = 13.0 min, 1% B.

All analyses in Assays 1 and 2 were performed in MS centroid, high-resolution mode. MS parameters were as follows: scan time of 0.3 sec, desolvation gas flow 900 L/h at a temperature of 350 °C, cone gas flow 50 L/h, source temperature 120 °C, capillary voltages in Assay 1: 3.2 kV in ESI+, 2.4 kV in ESI− and in Assay 2: 3.5 kV in ESI+, 2.7 kV in ESI−. All of the data were acquired using the lock mass to ensure accuracy and reproducibility. Leucine enkephalin was used as the lock mass at a scan time 0.5 s, interval 15 s and mass window ± 0.5 Da.

After statistical analysis, significant compounds were fragmented using the Fast Data Dependent Acquisition method, and the chromatographic as well as spectrometer parameters, except for the collision energy, were the same as for the MS mode. The resulting fragmentation spectra were compared to the spectra in the HMDB (Human Metabolome Database), [107] for putative annotation of compounds (level 2).

### 4.5. Metabolomic Data Preoprocessing and Normalization

The obtained LC/MS analysis files were loaded into Progenesis QI v3.0 software (Waters, Milford, MA, USA. The default parameter set for UPLC—High Res (Waters) was used for feature detection, retention time correction, alignment and putative annotation of compound classes—level 3 according to minimum reporting standards suggested by the Metabolomics Standards Initiative [108]. Afterwards, the raw intensity data from Progenesis QI v3.0 software were filtered: metabolite features with a blank contribution greater than 5% and with missing value above 50% (in a table with all features) as well as with a QC relative standard deviation (RSD) greater than 35% (in each batch separately) were removed. The remaining data were loaded to MetaboGroupS software (https://www.omicsolution.com/wukong/MetaboGroupS/ date of access 10 December 2021), for signal drift correction, batch effects elimination and normalization [109]. In MetaboGroupS software, missing values were imputed with the k-Nearest Neighbor (KNN) algorithm and for removing data skewness, a log2 transformation was applied. Subsequently, data were normalized using seven methods (median normalization, standard normalization, variance-stabilizing normalization, remove unwanted variation–random normalization, QC sample-based support vector regression, EigenMS and QC sample-based support vector regression). Coefficients of variation (CV) of entropy in QC samples with respect to no normalization and seven normalization methods in each analytical method are presented in Supplementary Material Figures S1–S4. For all our experimental datasets, the minimal CV was obtained from the EigenMS normalization method, and therefore, this method was applied [109].

### 4.6. Statistical and Chemometric Analysis

All analyses regarding general characteristics of the studied groups of workers were performed using Statistica (StatSoft, Tulsa, OK, USA) version 13.1 software for Windows. Prior to analysis, the data were tested for normality using a Shapiro–Wilk test. Statistical significance was established at *p* < 0.05. Determination of difference between groups was analyzed using one-way ANOVA followed by post hoc Tukey’s test for normally distributed data. Differences between pre- and post-shift urine Cr concentration were analyzed using a non-parametric Sign test for repeated measurements. Differences between controls and workers occupationally exposed to Cr(VI) were analyzed using a non-parametric Mann–Whitney test. A Chi-square test was conducted to compare smoking status and alcohol consumption in both subgroups.

In order to examine if the time of the sample collection from an employee influenced the metabolites profile, we performed a series of the paired difference test (Wilcoxon’s test) in order to identify if there were statistically significant differences between intensity of metabolites while comparing the same workers before (pre-) and after (post-) shift in pairs of each workers group: (A) pre- and post-shifts WW, (B) pre- and post-shifts WCP and (C) pre- and post-shifts WST. Hence, only the changes related to time and exposure that happened between these two time points were considered. Then, volcano plots were applied for verification if the metabolites between the pre- and post- shift groups of workers with large fold changes were also statistically significant. These scatter plots visualized the significance from a statistical test (by *p*-value) on the y-axis and the fold-change on the x-axis [110]. With scatter plots, it is possible to compare metabolite levels in different groups of workers. In the volcano plots, metabolites with a relatively low fold-change between the two compared samples are located near the center simultaneously, and metabolites with significant *p*-values (calculated from the relevant test) appear in the upper-right or upper-left side of the plot.

The PCA is a mathematical method, most commonly used for reducing the dimensionality of the analyzed data [111]. Using this approach, the new set of uncorrelated vectors of the original dataset is created and, on this basis, it is possible to analyze the similarities/dissimilarities of the studied data. In a nutshell, the PCA is based on the fact that some of the analyzed features (in this study, metabolites) describing the samples (OCC, WCC, post-shift WW, post-shift WST and post-shift WCP) are correlated with each other, and therefore, those metabolites include identical information on the sample. To overcome this problem and to choose the most important features during the PCA analysis, new, artificial variables such as principal components (PCs) are developed. PCs are linear combinations of original variables, and therefore, they are mixtures of initial features in different proportions [112,113,114]. As a result, the first PC (PC1) contains the largest possible amount of information (variance) in the original data matrix. Here, the PCA method was applied to the group of studied workers and controls (OCC, WCC, WW, WST and WCP) based on their metabolomic profile similarity and then to identify which particular metabolites were actually responsible for grouping of workers differently exposed to Cr(VI). Moreover, using the results of PCA, we also analyzed whether there were any trends between the metabolomic profiles of the workers and Cr concentrations in the urine as well as smoking, alcohol use declaration and the country where the workers were employed. We present the metabolomic profiles of the workers in the space of the 1st and 2nd PCs (PC1 and PC2 in the score plot) in accordance with the demonstrative criterion [115]. We assumed that the workers near to one another in the plot had a similar metabolomic profile. The physical interpretation of PC1 and PC2 was defined by the assumption that the contributions of metabolites with normalized loadings higher than module from 0.05 were significant. The PCA was performed in Python software3.

Classical univariate receiver operating characteristic (ROC) curve analyses were applied in order to identify potential biomarkers responsible for the grouping differentiation. Classical univariate ROC curve analysis is among the most frequently applied algorithms to generate ROC curves, to calculate area under the curve (AUC), to compute optimal cutoffs for any given feature, as well as to generate performance tables for sensitivity and specificity. The classical univariate ROC curve analyses were performed in MetaboAnalyst 5.0 platform (https://www.metaboanalyst.ca accessed on 10 December 2021).

## 5. Conclusions

Changes in urinary metabolite profiles from post-shift urine samples in Cr(VI) workers were clustered by type of industrial process (welding, chrome plating and surface treatment), distinct from controls and not explained by smoking status or alcohol use. We observed significant differences in the abundancy of metabolites primarily associated with fatty acid and monoamine neurotransmitter metabolism, oxidative modifications of the amino acid residues, the excessive formation of abnormal amino acids metabolites and changes in steroid and thyrotropin-releasing hormone. We putatively annotated a wide range of metabolites, with significantly enhanced signal intensity in workers exposed to Cr(VI) in comparison to the control group. The observed changes could also have resulted from work-related factors other than Cr(VI). Further targeted metabolomics studies are needed to better understand the observed modifications and explore the value of urinary metabolites as early indicators of adverse effects associated with exposure to Cr(VI).

## Figures and Tables

**Table 1:** Number of males exposed to Cr(VI) and males included in the control groups recruited from each country.

Country/ Groups	All Workers (*n* = 220)	WW (*n* = 101)	WCP (*n* = 61)	WST (*n* = 58)	All Controls (*n* = 102)	WCC (*n* = 33)	OCC (*n* = 69)
Poland	50	49	-	1	45	13	32
Portugal	45	3	7	35	24	2	22
Finland	46	23	18	5	21	6	15
Belgium	59	26	16	17	7	7	-
The Netherlands	20	-	20	-	5	5	-

Abbreviations: WW—welder, WCP—chrome plating; WST—other surface treatment or miscellaneous activities; WCC—within-company controls; OCC—outwith-company controls.

**Table 2:** General characteristics of the workers exposed to Cr(VI) and people included in the control groups (values are presented as mean ± SD or as percentage).

Parameter	All Workers (*n* = 220)	WW (*n* = 101)	WCP (*n* = 61)	WST (*n* = 58)	All Controls (*n* = 102)	WCC (*n* = 33)	OCC (*n* = 69)
Age	41.7 ± 11.0	39.1 ± 10.7	43.8 ± 12.2	43.9 ± 9.4	44.8 ± 10.1	44.0 ± 9.0	45.1 ± 11.0
Height (cm)	178.0 ± 6.4	177.7 ± 6.2	179.7 ± 6.5	176.6 ± 6.2	178.4 ± 6.9	180.7 ± 5.7	177.3 ± 7.1
Body mass (kg)	85.6 ± 13.6	85.1 ± 13.8	88.4 ± 15.1	83.6 ± 10.8	85.8 ± 13.0	91.5 ± 12.4	83.0 ± 12.4
BMI (kg/m^2^)	27.0 ± 4.0	26.9 ± 3.8	27.4 ± 4.8	26.8 ± 3.4	27.0 ± 3.8	28.0 ± 3.8	26.5 ± 3.7
Smoking status							
No (%)	52.1	59.4	37.7	54.5	75.2	81.3	72.5
Yes (%)	33.6	25.7	42.6	38.2	15.8	9.4	18.8
Former smoker (%)	14.3	14.9	19.7	7.3	8.9	9.4	8.7
Alcohol use							
No (%)	15.7	10.9	9.8	30.9	26.0	6.3	35.3
Yes (%)	84.3	89.0	90.0	69.0	74.0	93.7	64.7

Abbreviations: WW—welder, WCP—chrome plating; WST—other surface treatment or miscellaneous activities; WCC—within-company controls; OCC—outwith-company controls, BMI—body mass index.

## Data Availability

The data presented in this study are available on request from the corresponding author. The data are not publicly available due to privacy or ethical restrictions.

## Supplementary Materials

The following supporting information can be downloaded at: https://www.mdpi.com/article/10.3390/metabo12040362/s1, Figure S1: Coefficient of variation of entropy in QC samples (semi-polar and non-polar metabolite separations—Assay 1 in ESI+ mode) with respect to the other groups for the no normalization (original) and seven normalization methods (median normalization, standard normalization, VSN, RUV-random, QC-SVR, EigenMS and QC-RLSC). The minimum coefficient of variation is shown with a diamond symbol; Figure S2: Coefficient of variation of entropy in QC samples semi-polar and non-polar metabolite separations—Assay 1 in ESI- mode) with respect to the other groups for the no normalization (original) and seven normalization methods (median normalization, standard normalization, VSN, RUV-random, QC-SVR, EigenMS and QC-RLSC). The minimum coefficient of variation is shown with a diamond symbol; Figure S3: Coefficient of variation of entropy in QC samples (polar metabolite separations—Assay 2 in ESI+ mode) with respect to the other groups for the no normalization (original) and seven normalization methods (median normalization, standard normalization, VSN, RUV-random, QC-SVR, EigenMS and QC-RLSC). The minimum coefficient of variation is shown with a diamond symbol; Figure S4: Coefficient of variation of entropy in QC samples (polar metabolite separations—Assay 2 in ESI- mode) with respect to the other groups for the no normalization (original) and seven normalization methods (median normalization, standard normalization, VSN, RUV-random, QC-SVR, EigenMS and QC-RLSC). The minimum coefficient of variation is shown with a diamond symbol; Table S1: Putatively annotated metabolites.

## Appendix A

The HBM4EU Chromates study team consists of Kukka Aimonen^1^, Rob Anzion^2^, Radia Bousoumah^3^, Maurice van Dael^2^, Karen S. Galea^4^, Ivo Iavicoli^5^, Kate Jones^6^, Henriqueta Louro^2^, Sophie Ndaw^7^, Simo P. Porras^1^, Edna Riberio^8^, and Riitta Velin^1^ and Statistical team consist of: Natalia Buławska^9^, Dominika Jurkiewicz^9^ with the correspondent affiliations: ^1^ Finnish Institute of Occupational Health, 00250 Helsinki, Finland; ^2^ Human Genetics Department, National Institute of Health Dr. Ricardo Jorge (INSA), Toxicogenomics and Human Health (ToxOmics), NOVA Medical School, Universidade Nova de Lisboa, 1649-016 Lisbon, Portugal; ^3^ Comprehensive Health Research Center (CHRC), 1169-056, Lisbon, Portugal; ^4^ Institute of Occupational Medicine (IOM), Edinburgh EH14 4AP, UK; ^5^ Department of Public Health, University of Naples Federico II, 80131 Naples, Italy; ^6^ Health and Safety Executive (HSE), Harpur Hill, Buxton SK17 9JN, UK; ^7^ French National Research and Safety Institute, 54500, Vandoeuvre-les-Nancy, France; ^8^ Public Health Research Centre, NOVA National School of Public Health, Universidade NOVA de Lisbon, 1600-560 Lisbon, Portugal; ^9^ QSAR Lab Ltd., 80172 Gdansk, Poland.
