# Supplementary figures and images for: HBM4EU Chromates Study: Urinary Metabolomics Study of Workers Exposed to Hexavalent Chromium
Source: Metabolites. 2022 Apr 18;12(4):362. doi: 10.3390/metabo12040362 (PMC9032989; doi:10.3390/metabo12040362)

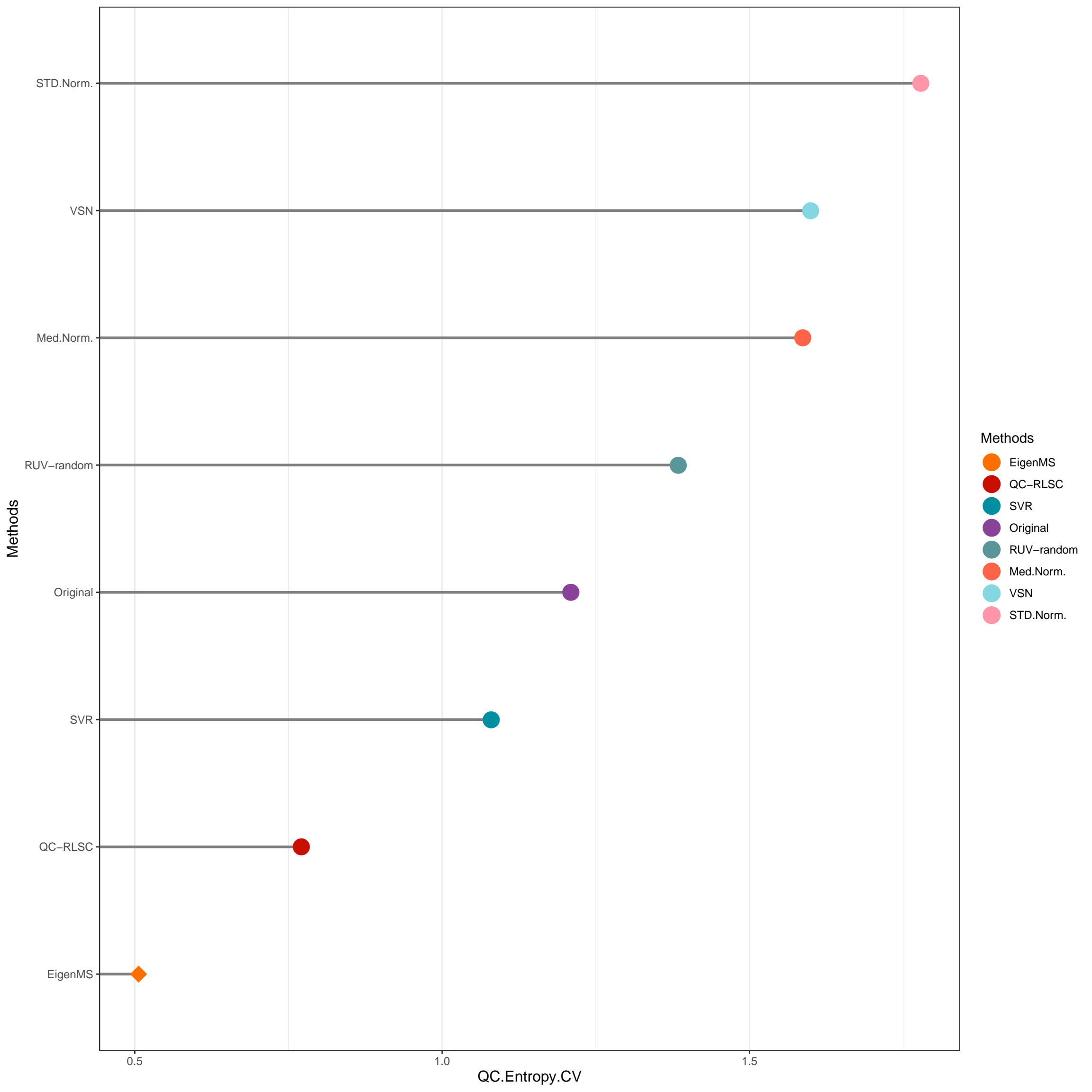

Supplement: Supplementary file 1 [file metabolites-12-00362-s001.zip › Figure S1. HSST3posResult.Summary_figure1630936035.19575.pdf]

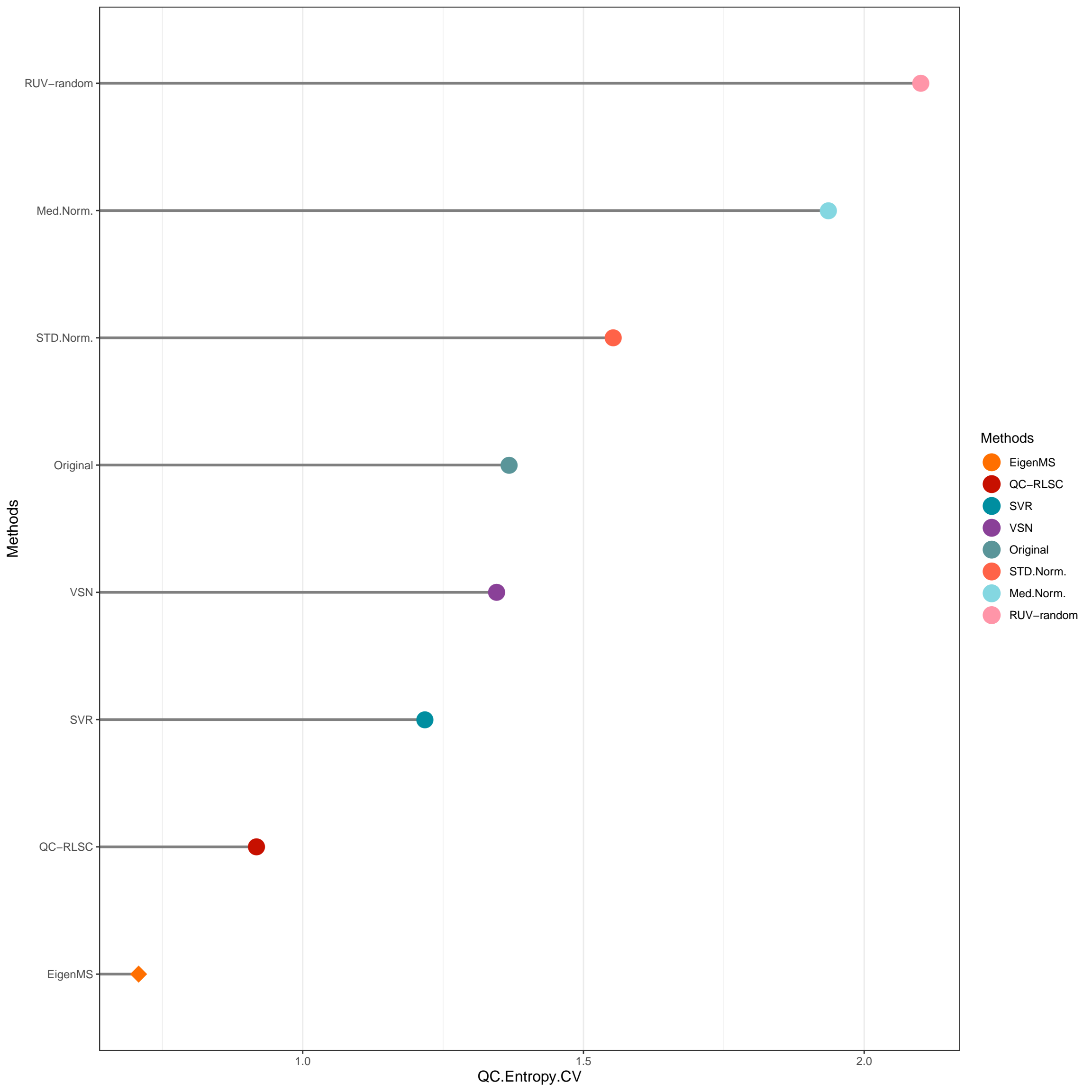

Supplement: Supplementary file 1 [file metabolites-12-00362-s001.zip › Figure S2. HSST3negResult.Summary_figure1630950289.39031.pdf]

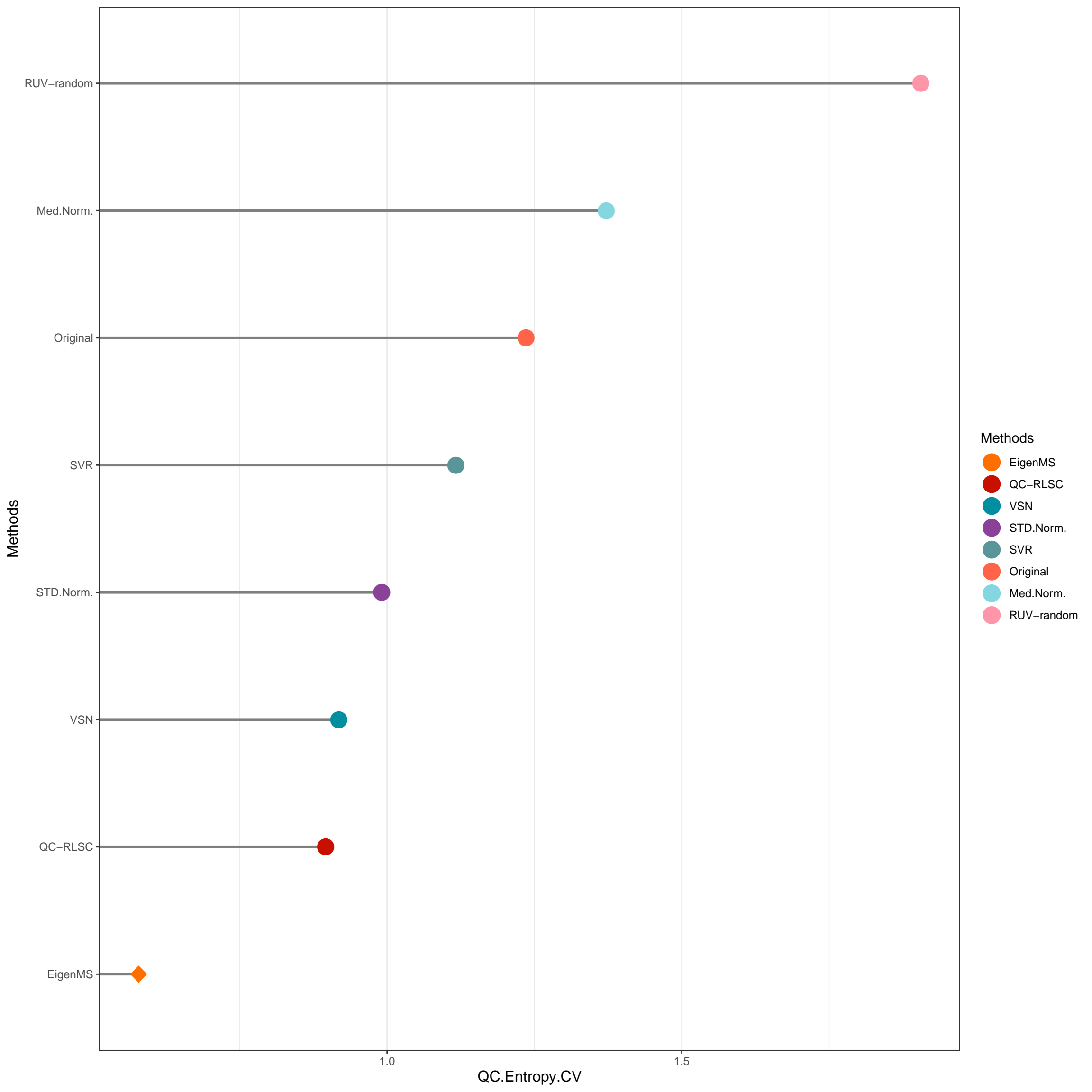

Supplement: Supplementary file 1 [file metabolites-12-00362-s001.zip › Figure S3. HILICposResult.Summary_figure1631033427.18674.pdf]

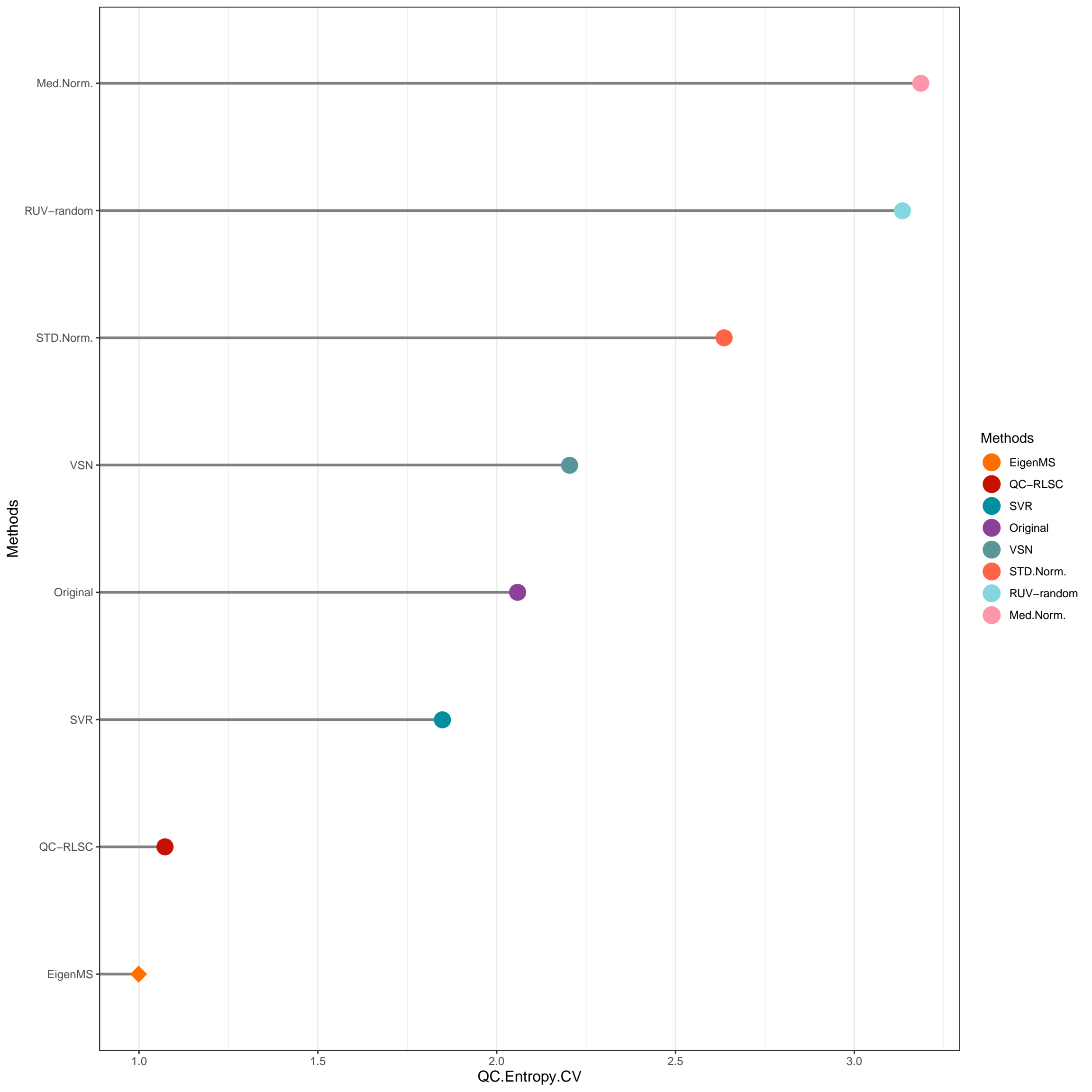

Supplement: Supplementary file 1 [file metabolites-12-00362-s001.zip › Figure S4. HilicnegResult.Summary_figure1637489760.06963.pdf]
